# 3D-conformal-intensity modulated radiotherapy with compensators for head and neck cancer: clinical results of normal tissue sparing

**DOI:** 10.1186/1748-717X-1-18

**Published:** 2006-06-21

**Authors:** Thomas G Wendt, Nasrin Abbasi-Senger, Henning Salz, Ines Pinquart, Sven Koscielny, Susi-Marie Przetak, Tilo Wiezorek

**Affiliations:** 1 Department of Radiation Oncology, Friedrich-Schiller-University Jena, Bachstrasse 18, D-07743 Jena, Germany; 2 Division Medical Physics of the Department of Radiation Oncology, Friedrich-Schiller-University Jena, Bachstrasse 18, D-07743 Jena, Germany; 3 Department for ENT Diseases, Friedrich-Schiller-University Jena, Lessingstrasse 2. D-07743 Jena, Germany

## Abstract

**Background:**

To investigate the potential of parotic gland sparing of intensity modulated radiotherapy (3D-c-IMRT) performed with metallic compensators for head and neck cancer in a clinical series by analysis of dose distributions and clinical measures.

**Materials and methods:**

39 patients with squamous cell cancer of the head and neck irradiated using 3D-c-IMRT were evaluable for dose distribution within PTVs and at one parotid gland and 38 patients for toxicity analysis. 10 patients were treated primarily, 29 postoperatively, 19 received concomittant cis-platin based chemotherapy, 20 3D-c-IMRT alone. Initially the dose distribution was calculated with Helax ^® ^and photon fluence was modulated using metallic compensators made of tin-granulate (n = 22). Later the dose distribution was calculated with KonRad ^® ^and fluence was modified by MCP 96 alloy compensators (n = 17). Gross tumor/tumor bed (PTV 1) was irradiated up to 60–70 Gy, [5 fractions/week, single fraction dose: 2.0–2.2 (simultaneously integrated boost)], adjuvantly irradiated bilateral cervical lymph nodes (PTV 2) with 48–54 Gy [single dose: 1.5–1.8]). Toxicity was scored according the RTOG scale and patient-reported xerostomia questionnaire (XQ).

**Results:**

Mean of the median doses at the parotid glands to be spared was 25.9 (16.3–46.8) Gy, for tin graulate 26 Gy, for MCP alloy 24.2 Gy. Tin-granulate compensators resulted in a median parotid dose above 26 Gy in 10/22, MCP 96 alloy in 0/17 patients. Following acute toxicities were seen (°0–2/3): xerostomia: 87%/13%, dysphagia: 84%/16%, mucositis: 89%/11%, dermatitis: 100%/0%. No grade 4 reaction was encountered. During therapy the XQ forms showed °0–2/3): 88%/12%. 6 months postRT chronic xerostomia °0–2/3 was observed in 85%/15% of patients, none with °4 xerostomia.

**Conclusion:**

3D-c-IMRT using metallic compensators along with inverse calculation algorithm achieves sufficient parotid gland sparing in virtually all advanced head and neck cancers. Since the concept of lower single (and total) doses in the adjuvantly treated volumes reduces acute morbidity 3D-c-IMRT nicely meets demands of concurrent chemotherapy protocols.

## Background

Intensity modulated radiotherapy (IMRT) by modulating the beam intensity (photon fluence) across each treatment field allows for better dose conformation to 3 dimensionally and particularly to concavely shaped contours of the target volume compared to conventional 3D conformal radiotherapy [1]. For fluence modulation several technical procedures have been developed: Static multileaf collimation, dynamic multileaf collimation, tomotherapy and physical compensators. In July 2001 3D-conformal intensity modulated radiotherapy (3D-c-IMRT) using metallic compensators was introduced in clinical practice at this institution. Methodological and technical optimization processes during the initial phase have been reported elsewhere [2].

Inverse dose distribution calculation algorithm is considered an indispensible characteristics of 3D-c-IMRT, which allows for optimization of fluence profiles to meet the prescribed doses for PTVs and critical normal tissues nearby to be spared. Improved parotid gland sparing has been demonstrated after inverse planning compared to traditional foreward planning [3].

This contribution deals with dose characteristics achieved in planning treatment volumes and normal tissue sparing particularly the parotid gland in 3D-c-IMRT for loco-regionally advanced squamous cell carcinoma requiring bilateral radiotherapy. The impact of different planning softwares and compensator characteristics due to changing materials used over the period will be analysed. Clinically the impact of parotid gland sparing on acute radiation induced morbidity will be investigated.

## Materials and methods

### Patients selection

3D-c-IMRT was used for patients with histologically proven squamous cell carcinoma of the pharynx, the larynx or oral cavity/floor of mouth treated either radically or postoperatively with curative intent. In all patients analysed 3D-c-IMRT was used for the entire treatment. Patients receiving only a part of their total dose by IMRT were not considered. Patients were selected not due to certain TNM-stages but due to likelyhood of irradiating a significant proportion of both parotid glands using standard techniques with consecutively high risk of chronic xerostomia. Nevertheless only advanced stages were treated. Patients with CUP syndrome received irradiation of the neck and the oro- and nasopharynx. Pretreatment evaluation consisted of a complete history and physical examination including endoscopy in unresectable cancers and detailed surgical and pathohistological reports of resected cancers, liver ultrasound and x-ray of the thorax. Loco-regional tumor extention was studied by MRT in all cases.

### Delineation of target volumes and normal tissues

Immobilisation of the head was accomplished by individually mounted light cast head and neck masks. Contiguous CT- slices (General Electric Lightspeed ^®^) of 5 mm thickness covering the primary and the neck without gap were imported into Helax ^®^-TMS. Intavenous contrast medium was given to better visualize macroscopic tumor if present. Contours were generated in all CT cross sections containing relevant information.

In all cases two different clinical target volumes were delineated: high dose volume (CTV1) harbouring high tumor cell burden e.g. macroscopic tumor or tumor bed after surgery of primary and/or lymph node metastases, and low dose volume (CTV2) assumed to contain low tumor cell burden e.g. adjuvantly treated regions of cervical lymphatic drainage. Since all tumors were in loco-regionally advanced stage adjuvantly treated neck regions included in all cases bilateral lymph node chains at levels I–V [4].

In order to create PTVs for dose distribution analysis margins surrounding the CTVs were added. To create the PTVs a generous concentric internal margin around macroscopic tumor or tumorbed of 5–10 mm towards all directions of the high dose CTV was given. Since preceding institutional investigations [unpublished data] showed small set up errors being of median 2 mm or less in three dimensions at the level of atlanto-occipital joint and of maximal lateral positioning error of 5.8 mm at the lower neck level a narrow additional margin to counteract for day-to-day set-up uncertainities was added. The parotid gland to be spared was generally nearby to the low dose volume. Therefore the margin around low dose CTV towards the gland was shrunken asymmetrically to minimize overlap with the gland's volume. High dose (surrounding CTV 1) and low dose (surrounding CTV 2) PTVs were contoured separately without common volumes but also without gap in between allowing for separate analysis.

In all cases the following normal tissues at risk (OARs) have been delineated and constraints were assigned for inverse planning: the parotid glands, the spinal cord with 5 mm safety margin, brain stem and more recently the glottic larynx without any safety margin. The mandible and oral cavity were not delineated routinely.

### Treatment planning and delivery

In patients with tumors of the nasopharynx, the oropharynx, the oral cavity or floor of mouth the gross primary or tumor bed as well as bilateral lymph node regions down to the level of hyoid bone were irradiated using the 3D-c-IMRT technique. The lower neck and supraclavicular fossae (regions III, IV, V B) were irradiated with a single anterior field. This has been done due to the immobilisation technique used which excluded shoulder fixation, expected reduced skin toxicity and reduced total doses at the lower neck in all cases except one. Only in case of gross tumor on both sides of the level of the hyoid bone 3D-c-IMRT portal arrangement extended down to the supraclavicular fossa. It was aimed to keep the median total dose at one parotid gland at 26 Gy or less. The parotid gland selected for protection was usually the one opposite the high dose volume. Thus a shallower dose gradient between low dose PTV and the parotid gland was easier to realize and the risk of underdosage at the high dose volume was minimized.

All 3D-c-IMRT treatments were performed by a standardized 7 portals arrangement. Fluence profile of each portal was modified by inserting a 3 D metallic compensator into the beam geometry of a linac Mevatron KD2 (Siemens Medical Solutions, Germany). Isodoses were generated by the inverse planning tools. In the initial phase the inverse planning tool of Helax ^®^-TMS software (Nucletron, Europe) was used with manual optimization, later the KonRad ^® ^software (Siemens Medical Solutions, Germany) to generate fluence profiles. The compensator fabrication process including dosimetric quality assurance procedures are reported elsewhere [2,5,6]. During this period two different metals were used for fabrication of compensators: initially tin granules embedded in wax were used for filling the 3 dimensionally cut styrofoam models, later MCP 96 was used. Concurrently to the substitution of tin wax compensators by MCP 96 alloy the planning software has also changed from Helax ^®^-TMS to KonRad ^®^.

### Fractionation

All treatments were given with 5 daily fractions per week. Dose prescription schedules were designed to perform a simultaneously integrated boost (SIB) especially in patients treated radically. Schedules empoyed were heterogenous at the initial phase, but finally single doses of 2.1 Gy for PTV 1 and 1.55 to 1.75 for PTV 2 were adopted as an institutional standard.

### Assessment of acute and chronic adverse effects

Acute und chronic toxicity was assessed semi-quantitatively by a specially trained radiation onoclogist (NA) according to the RTOG criteria. To allow patients to rate their subjective experiences with xerostomia a simple patient-reported questionnaire (XQ) was created according to questionaires reported from the literature [7,8]. Four questions address changes in month dryness, eating/swallowing, speaking, and sleeping function. To assess changes in salivation the patient was asked to mark on a scale from 0 to 6 (not difficult to extremely difficult) before radiotherapy has started, on the last day of radiotherapy and at 6 and 12 months after its completion. The questionnaire is given in detail in table 1.

**Table 1:** Xerostomia Questionnaire (XQ). Patients rated each item on a scale from 0 to 6; the higher the score, the worse they experienced their symptoms. 0: no complaints, 6: worst suffering.

*Due to dryness of your mouth/tongue and sipping liquids*

A-Rate your difficulties in eating and swallowing solid food.
B-Rate your difficulties in talking.
C-Rate your difficulties in sleeping.
D-In general, rate your difficulties during the daytime hours when not eating and not talking (oral comfort)

## Results

### Patients characteristics

From July 2001 until April 2005 44 patients were treated. 5 patients were excluded due to 3D-c-IMRT was restricted to the boost dose in the inital period. 39 patients are eligible for dosimetric analysis. 10 patients were treated for unresectable cancer, 29 after curative resection of tumor and neck dissection. Demographic and tumor characteristics are given in table 2. Due to a cardiac event one patient did not complete treatment. 38 patients are eligible for toxicity analysis.

**Table 2:** Demographic, tumor and treatment characteristics of 39 patients treated with 3D-c-IMRT using metallic compensators.

n	39
age (median, range)	57 (37–76) years
male : female	35 : 4
site of primary	
nasopharynx	4
oropharynx	20
oral cavity/tongue	9
hypopharynx/supraglottic larynx	5
CUP-syndrom	1
radical RT alone	10
postoperative RT	29
RT without simultaneous chemotherapy	20
RT with simultaneous cDDP	19

### Dose coverage of the parotid glands

**Figure 1:**
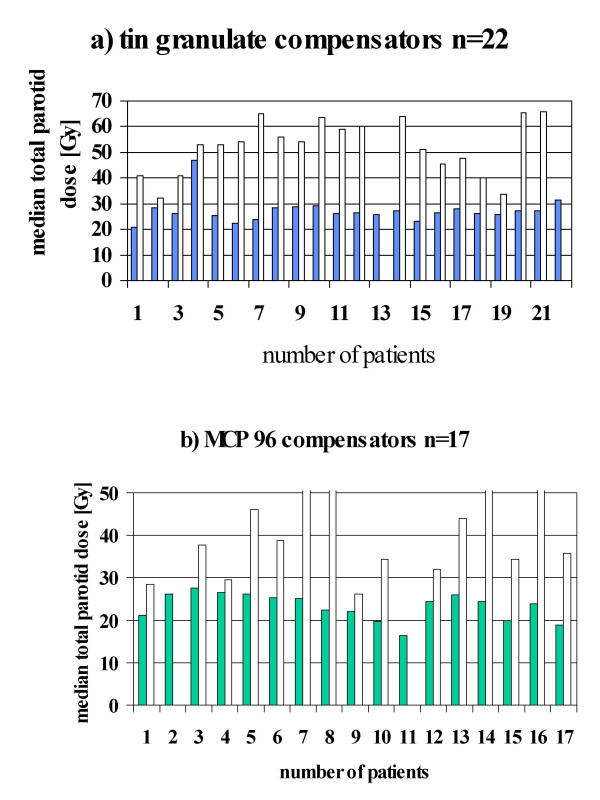
Median dose at parotid gland to be spared (solid bars) and not to be spared (open bars). (a) tin -granulate (b) MCP 96 Three cases: contralateral parotid not contoured.

With this method of 3D-c-IMRT a significant dose reduction in one parotid gland selected to be spared has been accomplished. The median dose at on gland could be restricted to 30 Gy or less in 37 of 39 patients treated and to 26 Gy or less in 29 of 39 patients. During the two periods different results have been obtained: with manually optimized planning with Helax ^® ^software and tin-granulate compensators (n = 22) median parotid gland doses ranged from 21–46 (mean 26) Gy (figure 1a, table 3). In two patients the doses were above 30 Gy, in one case due to very complex volumes to be treated. With fully automatized inverse KonRad ^® ^planning and MCP 96 alloy compensators (n = 17) with its larger dynamic range even better sparing could be achieved. In none of 17 patients the parotid gland dose exceeded 27.6 Gy, the lowest value being 16.3 Gy (mean 24.2 Gy) (figure 1b, table 3). However not only technology but also increasing experience may have contributed to improved parotid gland sparing. In all cases median doses at the parotid gland not planned to be spared were in the range above 26 Gy and may not have contributed to remaining saliva production (figures 1a and 1b).

**Table 3:** Dose coverage of the planning target volumes (PTV 1, high dose, PTV 2 low dose) and the better spared parotid gland after irradiation with tin-wax-compensators or MCP 96 alloy compensators in 39 patients with advanced head and neck carcinoma requiring bilateral irradiation.

Coverage high dose PTV 1 (D_95_/D_90_)	median 91%/97%
Coverage low dose PTV 2 (D_95_/D_90_)	median 92%/96%
	
Dose at spared parotid gland	mean 25.9 (16.3–46.8) Gy
Tin-wax-granulate	mean 26.4 (21 – 46.8) Gy
MCP 96 alloy	mean 24.2 (16.3–27.6) Gy

In cases when the high dose volume to be treated abuts both glands sparing becomes not sufficient. In one patient with a centrally located oropharyngeal cancer with bilateral large lymph nodes in regions II A/B right and in region IV left the parotid glands received median 46.8 Gy and 52.8 Gy. However this patient was treated in the early phase of the protocol with calculation procedure felt not optimal.

### Patient outcome

All 38 patients were followed until April 2006. During a median follow-up of 21 (11–44) months 6 recurrences in the high dose volume were encountered, 4 after primary chemoradiation, 2 after postoperative radiochemotherapy. 4 patients experienced recurrences within the low dose volume treated with 51, 49.5 and 54 Gy with single fraction size between 1.65 and 1.75 Gy. Two patients developed recurrences at the border between high and low dose volume. No recurrence at the border between the parotid to be spared and the contiguous PTV was observed.

### Acute toxicity

**Figure 2:**
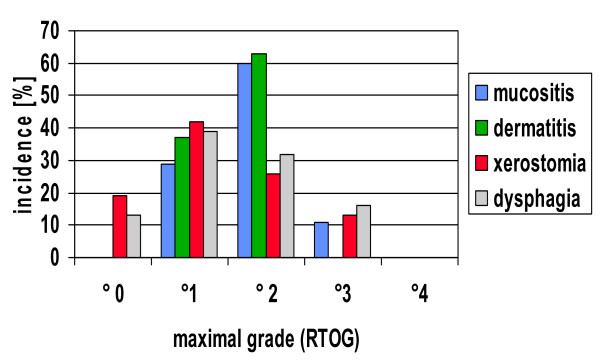
Maximal acute reactions observed. Grading according to RTOG classification.

Acute reactions were in general mild. Following acute toxicities were seen (°0–2/3): xerostomia: 87%/13%, dysphagia: 84%/16%, mucositis: 89%/11%, dermatitis: 100%/0%. No grade 4 reaction was encountered. It is noteworthy that despite high overall doses and a high proportion of patients treated with simultaneously given cis-platinum peak oral mucositis during therapy exceed grade 2 only in 4/38 (11%) (figure 2). At the end of therapy 88% of patients rated their xerostomia as °0–2 and 12% as °3.

Typically for the inherent field arrangement dermatitis showed an unconventional anatomical distribution pattern. Lips and perioral skin, which are often spared completely in conventional portal arrangement, showed mild dermatitis in virtually all patients. However the degree did not compromize treatment in any case. Due to posterior portals occipital hair loss occured regularly. 6 months after end of radiotherapy chronic xerostomia °0–2/3 was observed in 85%/15% of patients, none with °4 xerostomia.

## Discussion

One important issue of 3D-c-IMRT is normal tissue sparing. Among several critical tissues exposed when head and neck cancers are treated the parotid glands were of utmost interest. Chronic xerostomia is the most prevalent side effect. Patients rate it the most important factor of decreased quality of life after radiotherapy [9,10]. Overmore many symptomes will result like malnutrition, predisposition to fissures and ulcers, caries and infections after change of the composition of the oral flora being the most important [11]. Due to their ease detectibility in planning CT, their lateral position and their pairity they have been selected early when organ sparing by intensity modulated radiotherapy was introduced in clinical practice [12]. Tolerance dose of parotid glands is much lower than anticipated from earlier clinical experience [13]. Mean dose thresholds for unstimulated and stimulated saliva to reduce flow rates to less than 25% of the original rates were 24 and 26 Gy respectively [14]. Mean doses of 26 Gy at least at one parotid gland was recommended to preserve substantial saliva. However analyzing subjective responses the threshold seems to be lower. When IMRT yields median doses of 26 Gy or higher, a score measuring chronic xerostomia is reported slightly less favourable compared to doses of less than 26 Gy [15]. The range of doses below 26 Gy was analyized clinically in a small series. Patients reported higher oral comfort and less dry mouth complaints when the lower irradiated parotid gland received a median dose of 16 Gy or less compared to 22 Gy or more [16]. Decreasing radiation exposure to one parotid gland towards doses of 15 – 20 Gy has recenctly been shown to further help preserve saliva flow rates [17].

When this parotid gland sparing program was initated recently published data were not available and therefore the authors aimed at a median dose of 26 Gy at one parotid gland. This paper demonstrates that photon fluence modulation achieved by physical modulators is capable to result in parotid gland sparing. It has been documented that a fully automated inverse calculation algorithm along with improved modulator properties (MCP 96 alloy with its high dynamic range) further will reduce median parotid gland doses at 26 Gy. However it needs to be proven that this method is also capable to fulfill recently expressed figures of mean doses of 16 Gy, at least at one gland.

In this series 3D-c-IMRT results not only in parotid gland sparing but can decrease other clinically relevant toxicities. This is important, since acute toxicities remain a challenging issue particularly in intensified protocols e.g. incorporating modern chemotherapy schedules. In highly aggressive simultaneous chemoradiation regimen dose limiting acute mucositis grade 3 and 4 occur in up to 80% of patients [18]. Therefore 3D-c-IMRT with its low volume of heavily irradiated mucosa and large proportion of mucosa irradiated with low daily doses of 1.6–1.75 Gy seems an ideal technology to be combined with aggressive multidrug chemotherapy protocols.

However perioral dermatitis is a common finding due to ventral and ventrolateral portals very unusual in traditional techiques and seem to be independent from the type of 3D-c-IMRT technology used [19]. This acute reaction demand for more supportive care, however was not dose limiting in our series. Vice versa skin reactions at the cheeks are quite moderate due to a high number fo different dose entries.

It has been claimed that highly conformal radiotherapy may be an alternative to IMRT [20,21]. Particularly in cases where the target volume extends to base of scull e.g. in tumors of the tonsils and of the nasopharynx 3D-conformal radiotherapy without intensity modulation does not lead to significant dose reduction at parotid glands without changing the volume concepts. It may be an alternative for laryngeal and hypopharyngeal cancers without extension of the target volume towards to the base of the skull as far as it is in oropharyngeal cancers. Beside sparing of the parotid glands 3D-c-IMRT enables the planner to taylor isodoses also to spare other normal tissues, in particular the mandible, the larynx, pharyngeal muscles and probably also the pterigoid muscle. Reducing dose at these structures could result in reducing the risk for osteoradionecrosis as was shown in a early report [22]. The occurence of pharyngeal stenosis and fibrosis frequently seen in long term suvivors with advanced cancers seems to be more difficult, since these structures may be less easy to delineate especially in slim patients. However these are issues for future refinement of IMRT [23].

Although this paper was not intended to compare compensator technology with other IMRT technologies, advantages and disadvantages of the method presented may be discussed. Compensator technology allows for photon fluence modulation across a field of 32 × 32 cm maximum compared to leaf overtravel dependent smaller sizes (e.g. 20 × 20 cm) with some types of MLC. Compensator technology avoids tongue and groove or match line aspects. Its fluence properties are independent from multileaf collimator inaccuracies and therefore of higher reproducebility and lower time for individual QA measurements. Disadvantages of compensator technology are twofold: The need for accurate compensator fabrication demanding a skilled person and a programmable 3 D compensator cutter and the impossibility of a fully automatized performance of the treatment.

The technology presented allows for sufficient sparing of one parotid gland with median doses reported from series with step and shoot or sliding window technologies and gives therefore the opportunity to perform state of the art IMRT in head and neck cancer without using a multileaf collimator.

## Competing interests

The author(s) declare that they have no competing interests.

**Figure 3:**
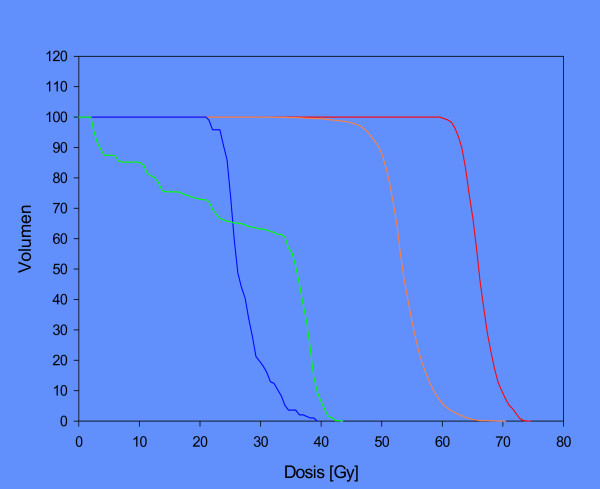
DVH of parotid glands: blue: to be spared, green: not to be spared.

**Figure 4:**
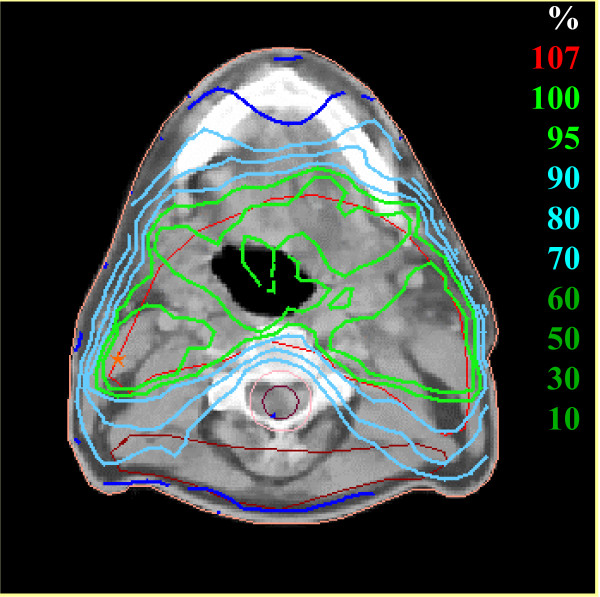
Dose distribution. Extensive left pharyngeal wall tumor and bilateral lymph node metastases pN2c.
